# Symmetry of the external acoustic meatus: A potential alternative reference plane for three-dimensional imaging in dentistry

**DOI:** 10.1016/j.heliyon.2024.e30460

**Published:** 2024-05-03

**Authors:** Fernanda Sanders-Mello, Ronald E.G. Jonkman, Josef Atay, Jasmine Atay, Frederik R. Rozema, Jan Harm Koolstra

**Affiliations:** a Academic Center for Dentistry Amsterdam (ACTA), the Netherlands; b Academic Center for Dentistry Amsterdam (ACTA), The Netherlands Department of Oral Medicine, University of Amsterdam, the Netherlands; c Department of Oral and Maxillofacial Surgery, Amsterdam UMC, University of Amsterdam, the Netherlands

**Keywords:** External acoustic meatus, Symmetry, Cone beam computed tomography

## Abstract

**Objective:**

In this study, we thoroughly analyzed how balanced the left and right sides of the external acoustic meatus are. Despite previous research focusing on the consistency of various anatomical features and the shape of the external acoustic meatus, which are important for creating guidelines to assess changes in the skull, there hasn't been enough attention given to how symmetrical it is. Our aim was to fill this gap by providing a comprehensive examination of its bilateral symmetry, which is crucial for accurate evaluations in dentistry and medicine.

**Study design:**

After importing 26 cone-beam computed tomography scans of patients into the ITK-SNAP 3D imaging software, a midsagittal plane was set up as the plane of symmetry for each patient. With this plane, we compared the positions of the most superior and inferior left and right points of the external acoustic meatus. We also compared the lengths and depths of the lines connecting the two points.

**Results:**

There were no statistically significant differences in the position, length, or depth of the external acoustic meatus between the right and left halves of the skull.

**Conclusion:**

Specific points on the skull, such as the highest (most superior MSP) and lowest (most inferior MIP) points, demonstrated a high degree of symmetry in the left and right halves. They demonstrated sufficient symmetry to establish a reliable reference plane. Along with the trajectory connecting them, these points can serve as viable alternatives to the Porion for three-dimensional imaging.

## 1 Introduction

When used in dentistry and orthodontics, cephalometric analysis depends on a reliable standard orientation and, thus, a coordinate system of the skull. The Frankfort Horizontal plane is often used as a reference for cephalometric analysis of sagittal radiographs [1]. This plane is defined by two landmarks: Orbitale and Porion. Orbitale is a projection of the inferior orbital margin, and Porion is the superior margin of the projection of both external acoustic meatuses (EAMs) [2,3]. For accurate localization of these landmarks, the patient faces the X-ray bundle perpendicularly while a lateral radiograph is obtained [4].

Given that the visible radiolucent opening of the EAM on a two-dimensional lateral cephalogram (Lat Ceph) results from the over-projections of various shapes and other cranial structures, the visibility of the anatomical Porion is limited. For this reason, Lat Ceph recordings in the literature also make use of the so-called machine Porion, which corresponds to a radiographic marker in the earplugs of the positioning adjustment equipment placed in the soft tissues of the EAM. However, this machine Porion is not a good substitute for the anatomical Porion [5] due to potential innacuracies introduced during image processing. CBCT machines use algorithms for image reconstruction, which can lead to distortions and artifacts.

The accuracy of lateral skull radiographs heavily depends on assuming symmetry between the left and right structures. However, issues like over-projection during imaging can introduce asymmetry, challenging this assumption and potentially affecting radiographic interpretation. The introduction of cone-beam computed tomography (CBCT) by Jacobs [6] has addressed this challenge. CBCT allows for the objective assessment of this assumption through detailed three-dimensional renderings of the skull's bony structures. This technological advancement not only extends cephalometric analysis into three-dimensional space but also emphasizes the importance of establishing an optimal three-dimensional reference coordinate system. Consequently, virtual orientation of CBCT images becomes crucial for enhancing the reliability of landmark detection, measurements, and segmentation in radiographic analysis. Moreover, this orientation relies on the construction of a reliable reference plane, such as the newly proposed Acta reference plane [7].

Landmarks similar to the Orbitale and Porion may be used to create a reference coordinate system for the skull for three-dimensional rendering by CBCT. While the superior margin of the EAM can be considered for the Porion, this may introduce an error because the EAM channel does not necessarily run horizontally from the external to the internal part of the meatus. Further, the left EAM is not necessarily a mirror image of the right EAM [8], as it has been observed to be conical, oval, or hourglass-shaped.

Despite previous research on the anthropometric features of the EAM, reports about the possible symmetry of its trajectory are relatively limited. However, Mizgiryte [9]reported trajectory asymmetry with respect to the midsagittal plane of the skull. The symmetry of the EAM is relevant for its use as a consistent structure in defining a reference plane, and CBCT scans can serve as viable alternatives to be used to analyze physical human skulls.

The superior and inferior points of the canal were used as reference points to determine the degree of symmetry of the EAM. Based on our hypothesis, we expected these points to demonstrate adequate symmetry for use as reliable reference points for establishing a reference plane for the skull to standardize the orientation and position of the images.

## 2 Materials and methods

This study was approved by the Ethics Review Committee of the Academic Center for Dentistry Amsterdam (ACTA) (reg. nr. 2022–94829) and conducted in accordance with the ethical principles outlined in the guidelines for research involving human participants. The study protocol, including the informed consent process, was reviewed and approved by the Ethics Review Committee of ACTA. Participants provided written informed consent before participating in the study. Twenty-six CBCT scans of living human skulls obtained for a study on the hypermobility of the temporomandibular joint were used in this study [10]. The age range of the participants was 18–65 years. The CBCT scans were obtained with a Newtom 5G scanner (QR Verona, Verona, Italy) and saved in the Digital Imaging and Communications in Medicine (DICOM) format. The exclusion criterion was the presence of major cranial bone abnormalities such as skull or jaw fractures. The CBCT settings for the scans were as follows: tube voltage, 110 kV; duration of exposure, 3.6 s; tube current, 38.25 (range, 22.35–55.76) mA; and voxel size, 0.3 × 0.3 × 0.3 mm.

The X, Y, and Z coordinates of both the MSP and MIP in the right and left cranial hemispheres were analyzed for the new coordinate system. Furthermore, we assessed the distance and angle of the line that connects the MSP and MIP (known as the MSP-MIP line) relative to the ACTA plane.

**Fig. 1:**
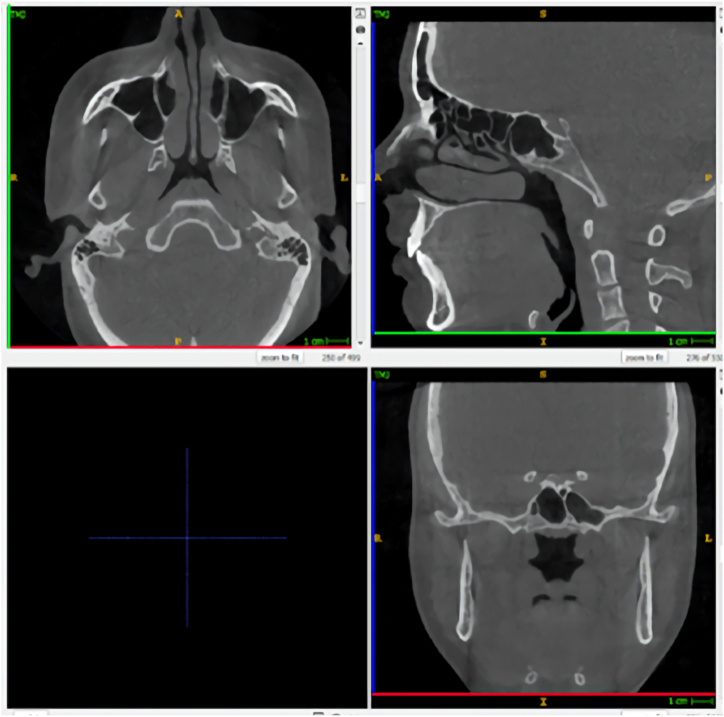
Depictions of sections in ITK-SNAP. Top left panel: axial sections, top right panel: sagittal sections, bottom right panel: frontal sections. Bottom left panel: reserved voor 3 D view (not applied). Green: sagittal axis; Blue: vertical axis; Red: transversal axis. (For interpretation of the references to colour in this figure legend, the reader is referred to the Web version of this article.)

All scans were independently analyzed by two senior master's degree students using ITK-SNAP 3D imaging software (http://www.itksnap.org). Serial axial, frontal, and sagittal sections were acquired for each scan (Fig. 1). All the coordinates found in these serial sections are listed in a database.

### 2.1 The ACTA plane

**Fig. 2:**
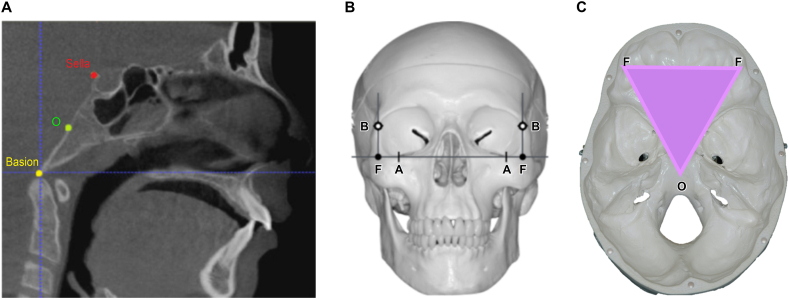
The three points defining the ACTA-plane. (A) Point O (green): midpoint between the dorsum sellae (red dot) and the Basion (yellow dot). (B) Construction of points F: the intersection of line A-A with the perpendicular line through the B line; point A (lowest point of the inferior orbital margins) and point B (most lateral point of the orbital margins). (C) ACTA plane axial view. (For interpretation of the references to colour in this figure legend, the reader is referred to the Web version of this article.)

We determined the symmetry of the EAM relative to the mid-sagittal plane. The latter was defined relative to the ACTA plane (Fig. 2A and B,C), which was defined by three points: the O-point; the midpoint between the Basion (the most anterior midpoint of the foramen magnum) and Sella (the most dorsocranial point of the dorsum sellae); and two F-points formed by the intersection of the horizontal line passing through the most inferior point of the orbit and the vertical axis line passing through the most lateral point of the orbit [7] (Appendix I).

### 2.2 Selection of anatomical landmarks

**Fig. 3:**
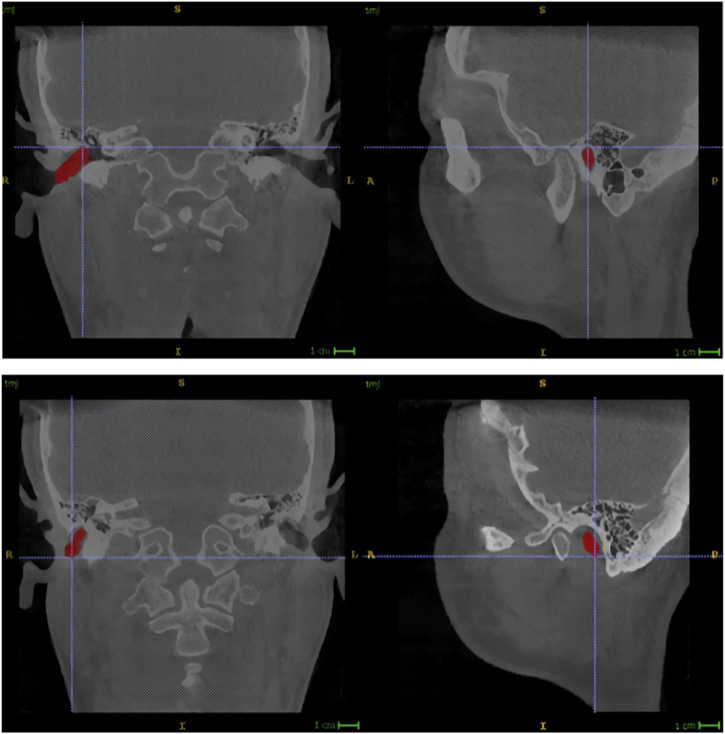
Reference points for the external acoustic meatus. Left panels: frontal sections, right panels: sagittal sections. Upper panels: location of the Most Superior Point (crosshairs). Lower panels: location of the Most Inferior Point (crosshairs). Red: segmentation of the external acoustic meatus. (For interpretation of the references to colour in this figure legend, the reader is referred to the Web version of this article.)

Our analysis of the symmetry of the EAM was based on two of its landmarks. The first was the Most Superior Point (MSP), which is often regarded as the anatomical Porion. We defined it as the superior point of the upper boundary of the bony portion of the EAM. On the CBCT scan, this point can be located by selecting the superior point in the frontal and sagittal serial sections (Fig. 3). The second landmark was the Most Inferior Point (MIP), which corresponds to the most inferior point of the lower soft portion of the EAM medial to the ear opening.

The MSP-MIP line represents the approximate trajectory of the EAM. Using GeoGebra (www.geogebra.org), we transformed the coordinates of these points in the CBCT coordinate system to those relative to the midsagittal plane.

### 2.3 Statistical analysis

IBM SPSS Statistics for Windows, version 27 (IBM Corp., Armonk, N.Y., USA), was used for the statistical analyses. A paired-sample *t*-test was used to analyze the X, Y, and Z coordinates of the MSP and MIP in the right and left cranial hemispheres for the new coordinate system; the distance of the MSP-MIP line; and the angle of the MSP-MIP line from the ACTA plane. Statistical significance was set at P < 0.05.

## 3 Results

**Fig. 4:**
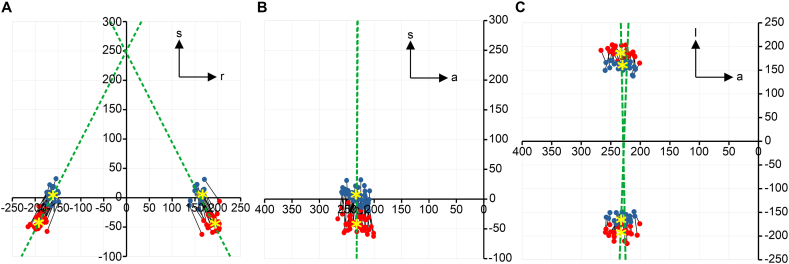
Reference points for the external acoustic meatus. (A) Frontal plane. (B) Sagittal plane. (C) Transversal plane. Blue dots: Most Superior Points (MSPs). Red dots: Most Inferior Points (MIPs). Yellow asterisks: means of the MSPs and MIPs. Dashed green lines: average direction of the MSP-MIP trajectory. Orientations: s: superior; r: right; l: left; and a: anterior. Coordinates in mm. (For interpretation of the references to colour in this figure legend, the reader is referred to the Web version of this article.)

The coordinates of the reference points and their relationships are shown in Fig. 4A (frontal plane), Fig. 4B (sagittal plane), and Fig. 4C (transverse plane). Four of the 26 CBCT scans were excluded from this study because the fields of view (FOVs) were not large enough to permit coverage of all the necessary points.

The mean differences in the position of the MSP between the right and left halves of the skull were as follows: 3.3 (SD: 10.7) voxels (1.0 mm) over the transverse axis, 0.4 (SD: 10.0) voxels (0.1 mm) along the sagittal axis and 0.2 (SD: 5.9) voxels (0.01 mm) along the vertical axis.

The mean differences in the position of the MIP between the right and left halves of the skull were as follows: 3.2 (SD: 8.9) voxels (1.0 mm) over the transverse axis, 0.7 (SD: 6.0) voxels (0.2 mm) along the sagittal axis and 0.7 (SD: 5.3) voxels (0.2 mm) along the vertical axis.

The mean lengths of the right and left MSP-MIP trajectories were 55.8 (16.7 mm) and 56.6 (17.0 mm) voxels, respectively. The mean difference between these lengths was 0.8 (SD: 5.0) voxels (0.2 mm).

The average angles between the right and left MSP-MIP trajectories and the ACTA plane were 56.0° and 56.5°. The mean difference between these angles was −0.5° (SD: 6.7°).

None of the above variables differed significantly between the right and left halves of the skull.

## 4 Discussion

We found no significant deviations in the symmetry of the selected landmarks. The shape of the EAM can be considered symmetric if the landmarks can be considered representative. Since the MSP corresponds to what we can consider the true anatomical Porion, it may be suitable for use as a reference plane for CBCT recordings.

It is challenging to mediolaterally delineate the (traditional) orbital landmarks of the skull because of the nearly horizontal orientation of the inferior orbital border. Therefore, the proposed method applies F reference points as a reliable alternative.

### 4.1 Accuracy of the midsagittal plane

We established the midsagittal plane as perpendicular to the line passing through the O-point, which is unequivocally part of the true midsagittal plane. However, our method treats the distance between the O-point and the plane based on the F-points as an approximation of the error. The average deviation we measured (1.7 mm) indicated that the plane we established may deviate from the real midsagittal plane by approximately 1.2°, which is clinically acceptable for orthodontic purposes in CBCT (Appendix II).

### 4.2 Direction of the EAM

We considered the MSP-MIP line representative of the orientation of the trajectory of the EAM. Given that both the MSP and MIP lie against the boundaries of this structure, the actual mid-sphenoid slope of the EAM is not necessarily equal to the MSP-MIP line.

### 4.3 Accuracy of the measurements

All points were determined independently. However, multiple measurements were not obtained for each anatomical landmark. Therefore, we cannot ascertain the intra- and inter-rater reproducibility of these points. The estimated error of the measurements was approximately 0.3 mm, which was the resolution of the CBCT scan.

### 4.4 CBCT orientation

**Fig. 5:**
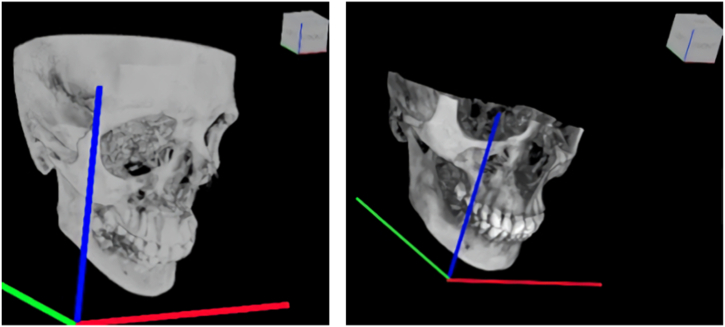
FOV setting: Left panel: maximum FOV. Right panel: limited FOV. Green: sagittal axis; Blue:vertical axis; Red: transversal axis. (For interpretation of the references to colour in this figure legend, the reader is referred to the Web version of this article.)

The axial, sagittal, and frontal serial sections ran along axes that defined the FOV of the CBCT scanner (Fig. 5). In the ITK-SNAP, the axes follow the RAI coordinate system (Right, Anterior, and Inferior). In the RAI model, the x-axis runs from right to left, the y-axis runs from anterior to posterior, and the z-axis runs from inferior to superior. The origin of the RAI coordinate system was the rightmost, most anterior, and most inferior position of the selected FOV. Given that it is not possible to position every individual in the same manner in a CBCT scanner, the position of the head relative to this zero point differs per scan, indicating that the RAI system is not a useful coordinate system for demonstrating symmetry between the right and left halves of the skull. To compare the right and left lines from the most superior point to the most inferior point of the EAM (MSP-MIP line), a new zero point, new sagittal axis, and new axial axis were determined for each patient and formed a midsagittal plane. This plane was used as a plane of symmetry, and it was convenient to place it in the middle of the head.

In this study, the ACTA reference plane [7] was useful. We assumed that the F-points were symmetrical and the line connecting them was a good transversal axis. The midpoint between the two F-points was used as the new zero point. The new sagittal axis was parallel to the ACTA plane and passed through the zero point. The new axial axis was perpendicular to the ACTA plane and passed through the zero point.

### 4.5 Limitations

This study was limited by its reliance on human CBCT scans to compare bilateral structures in a cohort with unspecified sex and age. This may have introduced biases. Therefore, the findings may not be generalizable to other populations. However, they serve as a foundation for future investigations into optimizing protocols, exploring advanced imaging technologies, and implementing dose reduction strategies. In future studies, a viable option would be to explore symmetrical and centrally located landmarks that will minimize the radiated area during medical imaging. This will reduce radiation doses to critical organs and enhance patient safety.

## 5 Conclusion

Analysis of selected anthropometric landmarks, such as the most superior point (MSP) and most inferior point (MIP) located in the acoustic meatus, as well as the trajectory that connects them, revealed significant bilateral symmetry. The results of this study corroborate the hypothesis that these specific landmarks are sufficiently symmetrical and can be used as reference points for determining a reference plane for the skull. Therefore, they can serve as alternatives to the Porion in three-dimensional environments. These findings have important implications for numerous fields, including craniofacial surgery and anatomical research, where precise measurements and analyses require accurate and reliable reference points.

A statement of informed consent.

Informed consent was acquired from all the patients and the patients consented to the publishing of all images, clinical data, and other data included in the manuscript.

## Data availability statement

Data associated with our study has not been deposited into a publicly available repository. Data will be made available on request.

## Funding

This research did not receive any specific grant from funding agencies in the public, commercial, or not-for-profit sectors.

## CRediT authorship contribution statement

**Fernanda Sanders-Mello:** Writing – review & editing, Writing – original draft, Validation, Methodology, Investigation, Formal analysis, Data curation, Conceptualization. **Ronald E.G. Jonkman:** Supervision, Investigation. **Josef Atay:** Investigation, Data curation. **Jasmine Atay:** Formal analysis, Data curation. **Frederik R. Rozema:** Project administration, Methodology. **Jan Harm Koolstra:** Supervision, Project administration, Formal analysis.

## Declaration of competing interest

The authors declare that they have no known competing financial interests or personal relationships that could have appeared to influence the work reported in this paper.

## Figures and Tables

**Fig. 6:**
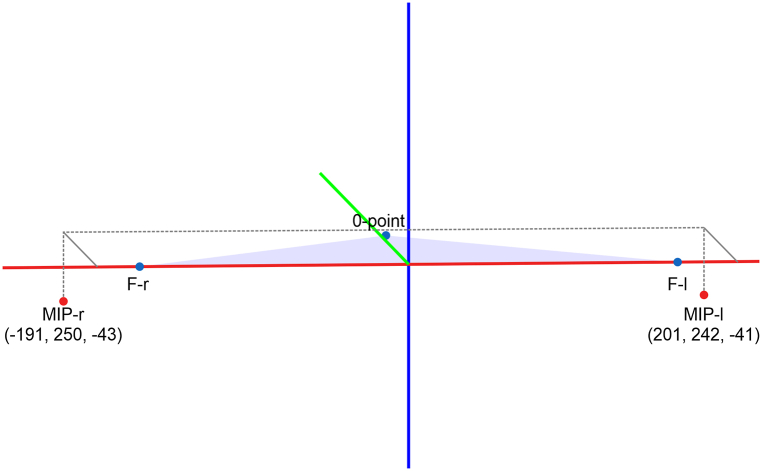
The new coordinate system developed in GeoGebra.

**Fig. 7:**
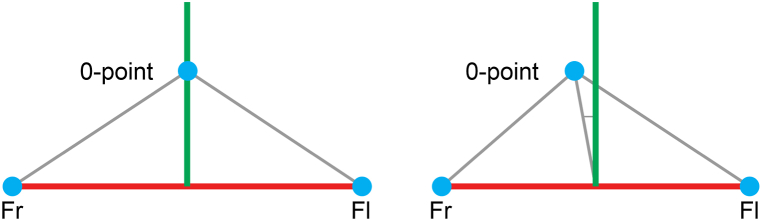
The ACTA-points form a triangle. The midsagittal plane in an asymmetric triangle

**Table:** 

Term	Description
Most Superior Point (MSP)	The highest point in a given context or structure.
Most Inferior Point (MIP)	The lowest point in a given context or structure.
External Acoustic Meatuses (EAMs)	The openings of the ear canal, located on either side of the head.
Fields of View (FOV)	The areas or regions that can be observed or monitored within a particular scope or perspective.
O-point	Origin point, a reference point often used as the starting point of measurements or calculations.
A-point	The most inferior point of the orbital margin on both the left and right sides.
B-point	The most lateral point of the orbital margin on both the left and right sides.
A Line	A line connecting the bilateral A-points, typically used as a reference line in anatomical or radiographic studies.
B Line	A line perpendicular to the A Line, passing through the B-point.
F-points	The intersection points of lines passing through the intersection of the A Line with the perpendicular line through the B Line.

**Table:** 

Landmark	Description
Orbitale	The lowest border of the orbit, typically seen as the lowest point on the orbital margin.
Porion	A-point on the skull located at the junction of the horizontal and vertical portions of the external auditory meatus. It is commonly used as a landmark in craniofacial measurements and studies.
Most Superior Point (MSP)	The superior point of the upper boundary of the bony portion of the external auditory meatus (EAM).
Most Inferior Point (MIP)	Corresponds to the most inferior point of the lower soft portion of the external auditory meatus (EAM) medial to the ear opening.

## Appendix I Construction of the F- points

The virtual F-points for the skull were defined as follows. From the frontal view of the CBCT scan, a line was constructed passing through the most inferior right and left midpoints of the orbital rim. Therefore this line is two-dimensional and linear (Z = ax + b). Points F1 for the right hemisphere and F2 for the left hemisphere were intersected by perpendicular lines (Z = −1/a * x + b) that pass through the most lateral points of the orbital rim. A graphing calculator was used to calculate the X and Z coordinates of the right and left F-points for each patient. The X and Z coordinates were entered in TK-SNAP. For the Y coordinate, 0 is provisionally assumed. The frontal serial section was slowly scrolled until the bone was reached, and the relevant Y coordinate was established (Fig. 6).

## Appendix II **Error analysis of ACTA-plane symmetry**

In an ideally symmetrical situation, the midsagittal plane runs through the O-point (Fig. 7). The distance of the computed O-point from the mid-sagittal plane was measured for each patient. The further the O-point was from the midsagittal plane, the more likely it was that the midsagittal plane between the two F-points was not a suitable plane of symmetry.
