# Supplementary material for: Symmetry of the external acoustic meatus: A potential alternative reference plane for three-dimensional imaging in dentistry
Source: Heliyon. 2024 May 3;10(9):e30460. doi: 10.1016/j.heliyon.2024.e30460 (PMC11096971; doi:10.1016/j.heliyon.2024.e30460)
Supplement: Multimedia component 1 [file mmc1.docx]

**Ethical Statement**

This study was approved by the Ethics Review Committee of the Academic Center for Dentistry Amsterdam (ACTA) (reg. nr. 2022-94829) and conducted in accordance with the ethical principles outlined in the guidelines for research involving human participants. The study protocol, including the informed consent process, was reviewed and approved by the Ethics Review Committee of ACTA. Participants provided written informed consent before participating in the study.
